# Selection of Water-Soluble Chitosan by Microwave-Assisted Degradation and pH-Controlled Precipitation

**DOI:** 10.3390/polym12061274

**Published:** 2020-06-02

**Authors:** Céline M. A. Journot, Laura Nicolle, Yann Lavanchy, Sandrine Gerber-Lemaire

**Affiliations:** 1 Group for Functionalized Biomaterials, Institute of Chemical Sciences and Engineering, Ecole Polytechnique Fédérale de Lausanne, EPFL SB ISIC SCI-SB-SG, Station 6, CH-1015 Lausanne, Switzerland; celine.journot@epfl.ch (C.M.A.J.); laura.nicolle@epfl.ch (L.N.); 2 Molecular and Hybrid Materials Characterization Center, Ecole Polytechnique Fédérale de Lausanne, EPFL STI MHMC MHMC-GE, Station 12, CH-1015 Lausanne, Switzerland; yann.lavanchy@epfl.ch

**Keywords:** low molecular weight chitosan, depolymerization, micro-wave assisted acid hydrolysis, water-soluble polymers, precipitation

## Abstract

In the field of gene therapy, chitosan (CS) gained interest for its promise as a non-viral DNA vector. However, commercial sources of CS lack precise characterization and do not generally reach sufficient solubility in aqueous media for in vitro and in vivo evaluation. As low molecular weight CS showed improved solubility, we investigated the process of CS depolymerization by acidic hydrolysis, using either long time heating at 80 °C or short time microwave-enhanced heating. The resulting depolymerized chitosan (dCS) were analyzed by gel permeation chromatography (GPC) and ^1^H nuclear magnetic resonance (NMR) to determine their average molecular weight (Mn, Mp and Mw), polydispersity index (PD) and degree of deacetylation (DD). We emphasized the production of water-soluble CS (solubility > 5 mg/mL), obtained in reproducible yield and characteristics, and suitable for downstream functionalization. Optimal microwave-assisted conditions provided dCS with a molecular weight (MW) = 12.6 ± 0.6 kDa, PD = 1.41 ± 0.05 and DD = 85%. While almost never discussed in the literature, we observed the partial post-production aggregation of dCS when exposed to phase changes (from liquid to solid). Repeated cycles of freezing/thawing allowed the selection of dCS fractions which were exempt of crystalline particles formation upon solubilization from frozen samples.

## 1. Introduction

Chitosan (CS) is a natural biopolymer composed of glucosamine and *N*-acetylglucosamine units, that gained the interest of scientists over the last four decades for its promises in pharmaceutics and medicine [1]. This polymer is produced on a commercial scale by the deacetylation of chitin, which is widely present in crustacea’s shells and fungi. The abundance of seafood waste represents a large reservoir of raw material for the production of CS, at low cost and in large quantities [2]. CS exhibits high biocompatibility as well as favorable adsorption properties and biodegradability [3,4], which make it a versatile biomaterial for the development of biomedical and pharmaceutical products [5]. For example, biomaterials based on CS display promising anti-bacterial and anti-oxidant properties that led to the development of dressing for wound healing [6]. Other applications use CS gels for drug-delivery systems and scaffold for bone treatment and biomedical imaging [3,7,8].

In the field of gene therapy, CS attracted much interest as a potential non-viral DNA vector. Most current strategies make use of viral vectors that can generate changes in the genome of the host cell with serious consequences [9]. Non-viral DNA, on the other hand, does not present the risk of uncontrolled genome insertion and is therefore considered safer than its viral counterpart. While naked DNA is unable to penetrate cells due to its high negative charge density, CS–DNA complexes assembled by electrostatic interactions were reported for cell transfection whilst protecting the nucleic acid from degradation [10].

The applications of CS are severely restricted by its poor solubility in the physiological environment and the lack of standardized characterizations. CS is only soluble in diluted acidic aqueous media and displays a complex behavior in solution such as macromolecular association (H-bond) [11] and polyelectrolyte effect [12], which is a challenge for characterization and its possible application as a functional material. The quality of CS should be described at least by the molecular weight (MW), polydispersity (PD) and degree of deacetylation (DD). Moreover, the purity level has a great influence on the biological characteristics of CS and CS derivatives, such as immunogenicity or biodegradability, but also has a profound effect on its solubility and stability [13]. Various commercial sources of CS are available, but specification data provided by the suppliers is often incomplete, which leads to misleading and inconsistent results [14,15,16,17]. Studies suggest that the properties of CS and its derivatives, in particular their antimicrobial properties, depend on the natural sources used for CS production (shellfish, fungi or bacteria) [18]. A refined conformational analysis reported by Weinhold et al. [19] on different CS preparations showed the absence of correlation between their conformation in solution with several parameters such as the number of acetyl groups, the pattern of acetylation and aggregation processes. This can be partially explained by the difficulties of fitting theoretical models to the non-standard behavior of CS [15]. The establishment of clear predictions is impeded by the varying characteristics of commercial CS preparations (such as solubility, DD, MW and PD), leading to results that might deviate from pharmacopeial standards. Despite the high promise of CS for biomedical applications, the current variability in the characteristics of commercial and further purified polymer sources represents a serious limitation towards its translation into the clinics.

The MW is considered to be one of the main factors determining the solubility of the polymer. Other parameters such as the DD or the pattern of acetylation are also expected to have an effect, although less pronounced [20]. Their impact on the solubility of CS preparations in aqueous solutions is currently strongly debated in the field [21,22,23]. The reduction of the MW of commercial CS is a common way to improve the polymer solubility and biological properties. Degradation methods include chemical depolymerization under oxidative, basic or acidic conditions, enzymatic treatment, or irradiation. Physical irradiation is less common, as it requires specific and expensive materials, extended reaction times and leads to an irregular degradation rate [24,25]. Similarly, the high cost of degrading enzymes is a considerable drawback of enzymatic treatment. In addition, their substrate specificity limits the range of the low MW which can be achieved [26]. Chemical degradation has been more widely studied and presents the most promising results [12,27,28]. Basic conditions are considered to reduce the DD faster than the MW, compared to acid hydrolysis of the 1,4-glycosidic bonds [29]. While lowering the MW of CS samples, oxidative degradations were reported to concomitantly lead to the loss of amino groups and the formation of carboxyls [30]. More recently, oxidative degradation (using oxygen peroxide) coupled with microwave treatment attracted considerable interest. This dual method has been shown to shorten reaction times, reduce the amount of oxidative agent needed and provide higher yields [31,32,33]. The factors influencing the depolymerization include the reaction time, the concentration of oxidative or acidic reagent, the quality and MW of the starting material and the liquid–solid ratio. However, uncontrolled chemical transformations (such as ring opening) as observed during oxidative degradation add to the list of undesirable unknown parameters of CS preparations and should be avoided.

For biological applications, the properties of CS are often further improved by chemical functionalization on the amino or hydroxyl groups. Despite an effort by the scientific community to improve the characterization for a given CS and CS derivative, chemical analyses are still lacking which complicates the evaluation of such chemical transformation. The extremely low solubility of CS in organic solvents and in pure water prevents the straightforward characterization by nuclear magnetic resonance (NMR) of the new grafted polymers, which is the most commonly used analytical technique to probe chemical transformations. Our work not only aims at investigating the production of depolymerized chitosan (dCS), but also the production of CS and CS derivatives soluble in water, a solvent suitable for NMR analysis and compatible with biological applications. 

Acidic conditions are considered to favor chain depolymerization over the removal of acetyl groups and reduce side reactions which are often associated with oxidative degradation [34]. We therefore studied the degradation of CS samples from two different suppliers in acidic conditions (HCl) and investigated the effect of microwave-assisted heating on the yield and characteristics of the resulting polymers. The characterization of the dCS preparations included the systematic determination of the MW (Mp, Mn and Mw), PD, DD and solubility in water, properties which are often partially described in previous publications. Other acidic reagents, such as nitrous acid, were reported for CS chain degradation and fractionation into neutral-soluble and neutral-insoluble dCS materials [35]. However, this method resulted in the formation of end-carbonyl groups, requiring an additional reductive step to prevent side-transformations on the highly reactive aldehydes. In addition, we herein report on the formation of dCS aggregates in solution, resulting from the removal of ions and sample freezing, and discuss the consequences of this change of crystallinity with regards to the discrepancies between the results reported in literature.

## 2. Materials and Methods

### 2.1. General Conditions

CS-A refers to the commercial CS from Tokyo Chemical International (TCI) with the following characteristics: η = 5–20 mPa·s (0.5% *w*/*v* in 0.5% acetic acid, 20 °C), DD = 84.5% (guaranteed ≥ 80%). CS-B refers to the commercial CS “for biochemistry” from Roth AG with the following characteristics: DD ≥ 90%. Depolymerized chitosan produced from CS-A and CS-B are referred to as dCS-A and dCS-B, respectively. 

The commercial sources for the following chemicals and consumables were: fuming HCl (37%), deuterium chloride (DCl): Sigma Aldrich; NaOH: Alfa Aesar; glacial acetic acid: Merck KGaA; sodium acetate trihydrate 99%: ABCR GmbH; deuterated water (D_2_O): Cambridge Isotope Laboratories, Inc. Dialysis membranes of regenerated cellulose (RC) of 3.5 and 7 kDa MWCO (Spectra/Por^®^ 3 and 1 respectively): Fisher scientific. Dialysis membranes of 14 kDa MWCO (Membra-Cel™, RC, packaged dry): Roth AG.

Aqueous solutions were produced with ultrapure water obtained from Milli-Q Integral System (Merck/Millipore, Schaffhausen, Switzerland). Centrifugations were performed with an Allegra X-30R centrifuge (Beckman Coulter, Nyon, Switzerland). pH monitoring during neutralization operations was done with a pH/Ion bench meter SC S220-K (Mettler-Toledo AG, Greifensee, Switzerland). 

### 2.2. Chitosan Depolymerization

The depolymerization of CS by conventional heating was conducted as follows. The polymer was dissolved over a 1 h period in 1M HCl to a final concentration of 1% *w*/*v*. The suspension was heated to 80 °C and then further stirred under reflux for 48 h. The solution was cooled to 30 °C before being neutralized to pH 7 using NaOH solution. The resulting light yellow suspension was centrifuged (10 min, 4700 ω, 20 °C) to isolate the liquid phase. Water was added to the precipitate up to the initial volume, shaken to obtain a homogeneous suspension and centrifuged a second time under the same conditions as above. The liquids were combined and transferred to a dialysis membrane (3.5 kDa MWCO), then dialyzed against water for a minimum of 3 days, changing the water three times per day and lyophilized. In case the solution was colored (dark yellow), it was filtered through a sterile 0.22 mm polyethersulfone (PES) membrane before lyophilization. In case of analysis of the precipitate, the precipitate isolated by centrifugation was suspended in water, dialyzed and lyophilized in the same way as the soluble fraction of dCS. 

The optimized conditions for the depolymerization of CS via microwave-assisted heating are described as follows. The CS was suspended in 1 M HCl to a final concentration of 1% *w*/*v*, typically 200 mg in 20 mL of solution. Commercial CS sources were dissolved in 1 M HCl solution, but required long hydration times (a few hours) or heating for approximately 20 min. In order to minimize acid-driven side reactions, CS (as a suspension) was transferred to a microwave-safe 30 mL vial and placed in the cavity of the microwave reactor (Monowave 400, Anton Paar). Video monitoring of the solution showed that the sample was entirely solubilized one minute after the start of the depolymerization program, at which point the CS solution had reached 60–70 °C. The optimal cycle of microwave depolymerization (under constant stirring of 300 rpm) followed the program below:

- Heat as fast as possible to 100 °C;
- Hold at 100 °C for 19 min;
- Cool down to 35 °C as fast as possible.

Once the resulting dCS solution reached a temperature of 30 °C, the solution was neutralized in two steps: 10 M NaOH solution was added until the solution reached pH 6.7. The suspension was left to equilibrate at room temperature for 40 min before being centrifuged (10 min, 4700 ω, 20 °C) to remove the precipitate. The pH of the liquid fraction was then further increased to 7.0 by dropwise addition of a 0.05 M NaOH solution. The white suspension was centrifuged a second time under the same conditions to remove the second precipitate, if any. The soluble dCS fraction was transferred to a dialysis membrane (3.5 kDa MWCO unless otherwise stated) and dialyzed against water for a minimum of 3 days, changing the water three times per day. In case of analysis of the precipitate, the precipitate isolated by centrifugation was suspended in water and dialyzed in the same way as the soluble fraction of dCS.

### 2.3. Evaluation of the Solubility of dCS in Acidic Aqueous Solutions and in DMSO

The solubility of dCS-A was first evaluated in aqueous acidic solution using 1M HCl. The solubility of the starting CS-A and CS-B were also evaluated for comparison. For each polymer, 30 mg were dissolved in 3 mL of pure water and left to dissolve for 15 min at room temperature under gentle shaking. The pH of the resulting suspensions was measured and indicated as starting pH. Then, 4, 8, 16 or 32 μL of 1M HCl aqueous solution were added and the resulting suspensions were again gently shaken over 10 min. The addition of 1M HCl and subsequent solution homogenization were repeated until a clear solution was obtained in each case. The pH was measured between each addition-shaking cycle and the last one corresponding to a clear solution was indicated as the final pH.

To test the solubility of the chitosan in DMSO, 20 mg of CS-A, CS-B or dCS-A were poured in 3 mL of DMSO, leading to a concentration of 6.7 mg/mL. The suspensions were gently shaken for 10 min and then left on the side for 10 additional minutes. A picture of the resulting solutions was taken (see Supporting information S-10, Figure S3).

### 2.4. Protocol for Complete Study of Post-Dialysis dCS Aggregation

CS-A was depolymerized in the microwave reactor at 100 °C for 19 min as described above. Once the solution reached 30 °C, 10M NaOH was added until the pH 6.7 was reached. The turbid solution was stirred for 45 min before being centrifuged (10 min, 4700 ω, 20 °C), and the aqueous phase was gently poured out to isolate the precipitate. The liquid phase was neutralized up to the pH 7 with a 0.05M NaOH solution and stirred for another 45 min. The solution was centrifuged a second time and the precipitate was discarded. The soluble dCS fraction was collected and dialyzed, at the end of which a new precipitate appeared in the dialysis membrane. After the isolation of this precipitate by centrifugation (named dCS (cycle 0)), the aqueous solution was divided in two equal volumes: one half of the volume was frozen whilst the second half remained at 25 °C overnight. The frozen solutions were then allowed to reach room temperature and centrifuged to collect the precipitate that had formed overnight before freezing the solution again. The numbering of the freeze–thaw cycle (cycle I, II, III, etc.) indicates how many times the dCS solution was subjected to freezing. All the samples went through a total of eight successive overnight freeze–thaw cycles and were centrifuged each time. No more precipitation was observed in the samples that remained at 25 °C over 20 days, or after four freeze–thaw cycles. Each precipitate was lyophilized and weighted individually. The concentration of dCS after each cycle was calculated by subtracting the mass of precipitate collected (after cycle I, II, etc.) to the mass of dCS (sol I). As a control, the concentration was also calculated by adding the amount of precipitate removed at each freeze–thaw cycle to the mass of dCS (sol II). Both calculations provided the same concentration of dCS in water, expressed in mg/mL. The protocol was performed four times and the presented values are the averages of four experiments.

### 2.5. NMR Analysis

^1^H-NMR spectra were recorded on a Bruker Avance III-HD spectrometer (Bruker, Billerica, MA, USA) at room temperature unless stated otherwise. The ^1^H frequency was at 400.13 MHz. The chemical shifts were reported downfield from tetramethylsilane. ^1^H signals were reported in ppm in pure D_2_O using the water’s residual signal at 4.79 ppm as the internal reference, or in a mixture of D_2_O + 1% DCl with the acetyl signal as a reference at 2.07 ppm. The shift of acetyl at 2.07 ppm corresponds to its position in pure deuterated water; we reported the same value in the spectrum recorded in D_2_O + 1% DCl. All the dCS samples were recorded in both solvents to compare the calculated DD values. The DDs calculated in D_2_O or D_2_O + 1% DCl were similar.

DD was calculated by comparing the integration of the protons from H3–H6 (4.00–3.49 ppm in D_2_O; 4.15–3.53 in D_2_O + 1% DCl,) set at a value of 5, and the relative integration of the acetyl signal (2.07 ppm, 3 protons). The percentage of the acetyl group in a given sample of dCS corresponded to the value of the integrated acetyl signal ($\int^{\;}Acetyl$) divided by 3. The DD can then be expressed as the following equation:

$$DD\;\left[\%\right]=\left(1-\frac{\int^{\;}Acetyl}{3}\right)*100$$

### 2.6. Gel Permeation Chromatography (GPC) Analysis

GPC analysis was performed on a PL-GPC 50 integrated GPC/SEC system (Agilent, Basel, Switzerland) equipped with a refractive index detector, a PSS NOVEMA MAX columns set (1× guard column, 10 μm; 2× analytical columns 1000 Å, 10 μm; 1× analytical column 30 Å, 10 μm), using 0.3 M aqueous AcOH and the 0.2 M aqueous NaOAc as eluents. The system was calibrated with pullulan from 180 to 1,220,000 Da. The samples of CS and dCS (2 mg each) were dissolved overnight in 1.5 mL of mobile phase. The solutions were filtered through a sterile 0.22 µm PTFE filter followed by injection (100 µL). The flow rate of analysis was 1 mL/min at 40 °C. The results of the GPC analysis were expressed as the number of the average molecular weight (Mn), the weight average of the molecular weight (Mw), and the molecular weight of the highest peak (Mp) and polydispersity index (PD).

## 3. Results

### 3.1. Acid-Mediated Depolymerization of Chitosan under Conventional and Micro-Wave Assisted Heating

**Figure 1:**
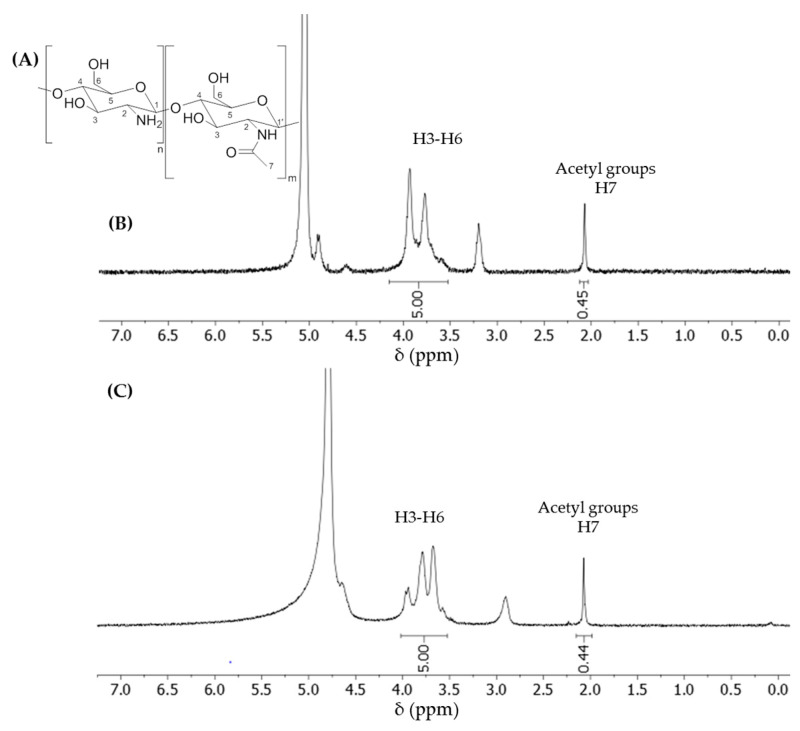
(**A**) Chemical structure of the partially acetylated chitosan. The typical ^1^H nuclear magnetic resonance (NMR) spectrum of (**B**) dCS-A in D_2_O + 1% DCl and (**C**) dCS-A in D_2_O, obtained by microwave-assisted depolymerization (Table 1, Entry 6).

We first compared the properties of dCS resulting from acid-mediated chain degradation under conventional or microwave-assisted heating (Table 1). In view of further functionalization and improved bioavailability in the physiological medium, this study focused on the characterization of the soluble fraction of dCS. Upon conventional heating, both commercial CS sources led to similar yields of depolymerization (Table 1, entries 3,4). Interestingly, only CS-A was degraded by acid-mediated depolymerization under microwave-assisted heating (Table 1, entry 6) yielding 15% of dCS-A after the optimization of the heating program and the reaction conditions. Variation on the reaction time between 15 and 23 min (at 100 °C) indicated an optimal yield for 19 min microwave-assisted heating (Supporting information S-4, Table S1). The effect of the temperature on the yield of the depolymerization was assessed from 80 to 180 °C (19 min reaction time, Supporting information S-5, Table S2). Below 100 °C, a very low yield of depolymerization was observed (2% yield), while heating over 120 °C resulted in the formation of degradation products (dark solids) which could not be efficiently separated from dCS-A. The optimal acid-mediated chain degradation temperature was determined at 100 °C, resulting in a reproducible yield and the high purity of dCS-A. Finally, the concentration of the starting solution was varied from 0.5 to 6 wt % (Supporting information S-6, Table S3). Above 2 wt % initial concentration, the internal pressure inside the reactor significantly increased during the process, leading to the interruption of the microwave heating program over 10 bars (4 and 6 wt %) and the formation of degradation by-products. An initial concentration of 1 wt % is thus recommended for the microwave-assisted depolymerization. While conventional heating was invariably accompanied by uncontrolled degradation resulting in colored solutions and solids, the dCS-A obtained by microwave-assisted depolymerization (1 wt %, 100 °C, 19 min) was exempt of degradation side-products. A significant decrease in the PD value (from 2.82 to 1.41) indicated the improved homogeneity of dCS-A, as compared to the starting commercial source. In addition, the ^1^H NMR analysis of dCS samples revealed that the conventional depolymerization over 48 h produced fully deacetylated polymers, whilst microwave-assisted depolymerization maintained the DD of the starting CS sources (Table 1, entries 5,6 and Figure 1), suggesting that the acetyl groups were sensitive to the extended exposure to acidic conditions. Surprisingly, CS-B proved resistant to microwave-assisted depolymerization with less than 1% yield recovered under all the tested conditions (including an extended reaction time up to 3 h and heating up to 120 °C, see Supporting information S-7, Table S4), but not to conventional heating (49% yield, similar to dCS-A). A high DD was reported to be correlated with a higher degree of crystallinity, which was also associated with a higher resistance of the 1,4-glycosidic bond to breakage [36,37]. In this respect, one might hypothesize that the depolymerization of CS-B needs extended heating time to be initiated (up to 3 h reaction time, only a trace amount of dCS-B could be recovered). However, a longer reaction time was not attempted as the fast production of dCS samples was targeted. 

In order to evaluate the effect of depolymerization on the solubility of the resulting polymer dCS-A, we performed a progressive dissolution of CS-A, CS-B and dCS-A in H_2_O (10 mg/mL) using an aqueous solution of 1M HCl. The pH needed to reach full solubility for each polymer and was determined and referred to as the final pH (Table 2). The starting pH of each batch was almost neutral and similar, except for CS-B whose pH value reached 7.48 (entry 2). This difference was attributed to the higher degree of deacetylation characterizing CS-B (99%), as compared to CS-A and dCS-A (85%). The ratio of free amines in CS-B was thus higher than for the CS-A leading to a higher pH in pure water as well as a better buffering capacity. The solubility of chitosan was clearly improved after the depolymerization process (entry 3 vs. entries 1 and 2), allowing downstream operations in almost pure water (full solubilization at pH 5.97, 10 mg/mL). The solubility of dCS-B was not evaluated as the yields of depolymerization were very low (*cf*. Table 1).

Polar organic solvents were also of interest with regards to CS functionalization, involving the nucleophilic amines and primary alcohols of the polysaccharide backbone. The solubility of dCS-A was therefore evaluated in DMSO at a concentration of 6.7 mg/mL (22 °C) which is commonly encountered when running reactions with CS (Supporting information S-10, Figure S3). While CS-A was completely insoluble in DMSO, forming aggregates that could not be dispelled, dCS-A was homogeneously dispersed, resulting in a fine stable suspension suitable for chemical transformations. CS-B was suspended upon vigorous stirring in DMSO. However, powder deposition was observed as soon as the stirring was stopped.

### 3.2. Molecular Weight Selectivity

As presented earlier, the high variability of the size of a given CS sample was an issue for pharmaceutical applications. The fine selection of the MW range, expressed as the PD, is one of the most significant parameters to qualify the homogeneity of the polymer. We identified several parameters that allowed to select a certain MW during the purification process.

We investigated the effect of the pore size of dialysis membranes on the collected MW and PD of dCS, obtained from CS-A by microwave-assisted depolymerization. A large batch of dCS was divided into three equal volumes for subsequent dialysis using 14, 7 and 3.5 kDa MWCO membranes (Table 3).

The GPC values indicated that the dialysis membrane with 14 MWCO provided a polymer about 13% bigger than with a 3.5 MWCO membrane. The use of a smaller MWCO slightly increased the yield but decreased the MW homogeneity of the product (higher PD values). This small increase in the PD index was consistent with the size of the membrane’s pores. As the long dCS chains remain within the dialysis pocket for all the MWCO size, the length of the dCS being retained in the dialysis pocket decreases with the MWCO of the membrane, hence enlarging the scope of smaller membranes. It is to be noted that the Mw values from Table 3 present a variation of about 5% (average Mw = 19.4 ± 1.0 kDa), which is likely to arise from the error range of the GPC (see Supporting information S-8, Table S5 and Figure S1) and from the structural pore difference between the membranes. 

We next investigated the effect of the pH at which the crude dCS solution was neutralized and the speed at which the given pH was reached (Table 4). Both parameters were shown to influence the MW and the yield of the final polymer. The selection of the soluble fraction at pH 7.4 led to an average MW approximately 50% smaller (entries 5 and 6). Stepwise neutralization, including a 45 min waiting time before centrifugation, allowed to improve the yield by approximately 5%. It was determined that the removal of the precipitate that accumulates when the solution is neutralized to pH 6.7, followed by the further neutralization to pH 7.0 and the selection of the soluble dCS fraction was the optimal protocol.

It was theorized that a gradual change in pH (from 1.0–1.5 to 6.7, then from 6.7 to 7.0 or higher) limited the premature precipitation of low-MW dCS by slowing down the formation of dCS aggregates and giving the suspension time to stabilize. It is to be noted that no further precipitation was observed when the pH was increased between 7.4 and 12. These data also highlighted that the collection of the soluble fraction between the two pH values allowed the selection of intermediate MW and a decrease in the PD value as the pH increased above 7.0 (Table 4 and Supporting information S-9, Figure S2).

To our knowledge, the separation of CS MW fractions by GPC was reported only by Weinhold et al. [19], but was restricted to the collection of small samples (a few mg) for analysis. This method is not suitable for the isolation of samples for downstream chemical functionalization and further biological evaluation. Separations by sequential pH precipitation and dialysis/lyophilization allowed for larger batches to be collected (up to 50 mg at each run) with a PD ≤ 1.5. 

### 3.3. Solubility of Depolymerized Chitosan

Very few scientific studies discuss the variation in the crystallinity of CS. In few cases, aggregates were detected in acetic acid solution by light scattering [38], scanning electron microscopy [39] and capillary zone electrophoresis [40]. While aqueous acetic acid solutions are generally accepted as good solubilizing media for CS, these findings indicate that non-detected nanoscale CS aggregates might have been used for in vitro and in vivo investigation thus far. In an effort to study and limit the occurrence of such dCS nanoparticles, we investigated the formation of dCS precipitate in water. During the purification of dCS-A resulting from microwave-assisted depolymerization, it was observed that a small fraction of the polymer precipitated during dialysis. We wondered whether this aggregation was solely dependent on the removal of NaCl ions (resulting from post-depolymerization neutralization), or was resulting from a non-equilibrium state of dCS in solution.

We first investigated the solubility of desalted, lyophilized dCS-A in pure water and in water + 1% *w*/*v* NaCl, final concentration 0.1 mg/mL. After 48 h of continuous stirring at 25 °C, the two samples showed equal amounts of aggregates still present in solution. This suggests that the presence of NaCl ions in a neutral solution has no effect on the solubility of the polymer. The apparent reduction of solubility of lyophilized dCS-A (compared with non-lyophilized dCS-A) suggested that the crystallinity of the polymer changes during lyophilization, resulting in insoluble aggregates or insufficiently solvated residues of polymer in solution. The diminution of solubility resulting from sample freeze drying is very rarely mentioned in literature, possibly because in most work, slightly acidic aqueous solutions are used.

**Figure 2:**
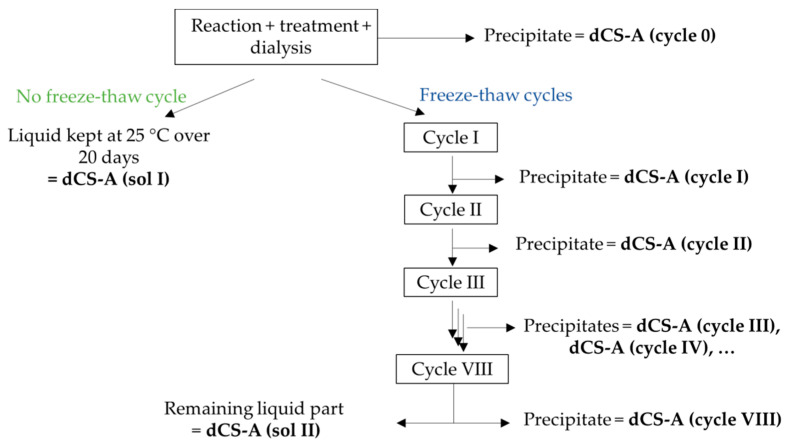
Diagram of the freeze–thaw cycles protocol. Names of the samples as reported in Table 4 are indicated in bold.

We next investigated the aggregation process in more detail (protocol 2.4, Figure 2). The soluble fraction of dCS-A produced by microwave-assisted heating was dialyzed, at the end of which a precipitate started to appear in the dialysis membrane. After the removal of this precipitate (named dCS (cycle 0)), the solutions were divided into two equal volumes: one half of the volume was frozen whilst the second half was left in solution at 25 °C. The frozen solutions were then allowed to reach room temperature and then centrifuged again. The precipitates were collected before freezing the solution again. The numbering of the freeze–thaw cycle (cycle I to VIII) indicates how many times the dCS solution was subjected to freezing. All samples went through a total of eight successive overnight freeze–thaw cycles. No further precipitation was observed for the samples that remained at 25 °C, over 20 days, or after four freeze–thaw cycles.

Table 5 presents the amount of precipitate removed at each freeze–thaw cycle expressed as a decreasing concentration of solubilized dCS. This study revealed that dCS-A, in pure water solutions, exposed to freeze–thawing does not aggregate below a concentration of 0.15 mg/mL. Decreasing the concentration of the sample to 0.1 mg/mL after the first freeze–thaw cycle did not remove the precipitate, suggesting that the formation of aggregates is irreversible in water. On the other hand, no precipitation was observed after 20 days for samples that were kept at 25 °C and at a concentration of 0.22 ± 0.02 mg/mL. We postulate that the liquid–solid phase transition may be responsible for the reorganization of the intra- and inter-molecular interactions, hence changing the crystallinity of the polymer [41]. 

A complementary study was performed on dCS-B resulting from microwave-assisted depolymerization (120 °C, 60 min), despite the limited quantity of polymer recovered after chain degradation. The soluble fraction was separated into two equal volumes prior to dialysis. One half was lyophilized directly after dialysis (dial-dCS-B), the second half was centrifuged to isolate the solid particles (prec-dCS-B) from the liquid phase (liq-dCS-B). GPC results are presented in Table 6.

**Figure 3:**
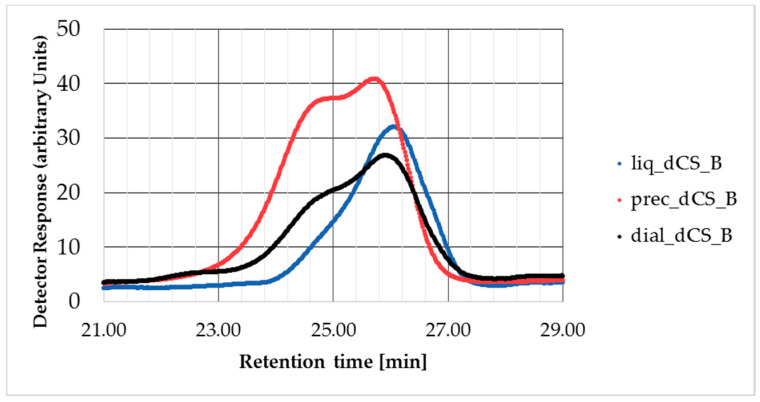
Eluogram of dCS-B between 21 and 29 min of elution time. Black: dial-dCS-B (corresponds to the two other traces combined); red: prec-dCS-B; and blue: liq-dCS-B.

The results indicated that the removal of the precipitate from the soluble fraction allowed the selection of dCS with a smaller MW and PD in the soluble fraction. The presence of fully solubilized and solid dCS-B chains in solution is supported by the feature of the GPC eluograms (Figure 3). The trace of dial-dCS-B showed two local maximums at 24′43″ and 25′40″ approximately. By comparison, the traces of prec-dCS-B and liq-dCS-B revealed that the two populations of dCS-B chains can be discriminated by the separation of the aggregates from the soluble phase. The smaller dCS-B chains remained in solution, as observed by a single maximum at 26′8″. The trace of prec-dCS-B maintained its double maximum. We postulated that this was the result from the method of separation of the precipitate, during which some of the solution remained entrapped in the precipitate. Similarly shaped traces have been reported during a DLS analysis of CS aggregates in solution by Anthonsen et al. [42], which was also interpreted as the presence of crystalline dCS chains in solution.

## 4. Discussion

The depolymerization of CS under microwave-assisted acid-mediated chain degradation with subsequent stepwise neutralization and centrifugation allowed the selection of the pure water-soluble fraction of dCS and significantly improved the PD of the resulting polymer. This degradation method produced dCS with a highly reproducible MW (less than 5% standard deviation over four batches) (Supporting information Table S5 and Figure S1). The full solubilization of dCS-A (10 mg/mL) in aqueous solution was obtained at pH 5.97, while acidification up to pH 2.19 was necessary to solubilize CS-A. We showed that the microwave-assisted heating shortened the reaction time of the acid-mediated depolymerization, which resulted in the limited uncontrolled degradation. This was supported by the obtention of colorless solid dCS and dCS solutions. The limitation of side-deacetylation, due to a shorter exposure to acidic conditions, is another advantage of the microwave-assisted depolymerization. We also demonstrated that the PD index and size of the dCS can be tuned by preforming a stepwise neutralization involving the removal of intermediate precipitates and selecting dialysis membranes of different molecular weight cut-off. In addition, while both commercial CS materials could not be dispersed in any polar organic solvent, dCS-A formed a homogeneous fine suspension in DMSO (6.7 mg/mL), which is a prerequisite for further chemical transformations. 

It is to be noted that the data presented in this study were obtained using the same batch of starting material for CS-A and CS-B. However, we noticed that the physicochemical properties of CS (from a same supplier) could change from one batch to another. In particular, the DD was observed to vary between 85% and 76%. While there is very limited options at present to reduce the variations in the characteristics of commercial CS sources, the chain degradation and selection of dCS fractions soluble in pure water appears as an appealing strategy to ensure the reproducible physicochemical characteristics of CS preparations for further downstream functionalization and biomedical applications. 

## 5. Conclusions

CS is known for being a highly valuable biopolymer candidate for medical and pharmaceutical applications. However, we believe that the scientific community needs to increase awareness concerning the lack of characterization and standardization of this polymer. The most obvious challenge of CS relies in its very low solubility in organic solvents and neutral aqueous solutions. Its solubility can be increased by degrading the CS chains into smaller, depolymerized polymer chains (dCS). Depolymerization in acidic conditions (1M HCl) was studied either using conventional heating or microwave-assisted heating. The use of the microwave proved to be superior to conventional heating as it reduced the reaction time from 48 h to 19 min whilst preserving the *N*-acetyl groups on the polymer backbone and preventing degradation. Our results suggest that the depolymerization in acidic conditions needs to be adapted to the CS of choice, as the behavior of this polymer changed with the source (and batch) of the commercial source. Although we cannot comment on the kinetics of the 1,4-glycosidic bond breakage, we postulate that the very low amount of acetyl groups in CS-B is the determining factor for its apparent resistance to chain breakage. A high DD was linked to a higher crystallinity of CS [36,43], which reduces the surface of the polymer available for nucleophilic attack and the likelihood of complete 1,4-glycosidic bond breakage [37]. 

Beyond the demonstration that microwave-assisted depolymerization provides a fast access to dCS preparations which are fully soluble in pure water, we also highlighted that the average MW of dCS can be selected by isolating soluble fractions at pH between 6.6 and 7.0. The higher the pH at which dCS is collected, the smaller the MW will be. In the neutralization process, precipitation was observed from pH 6.7, increased up to pH 6.9, and then reduced linearly up to pH 7.4. From pH 7.0, almost no precipitate was formed anymore. The selection of the soluble fraction at each step of the precipitation process allowed the highly reproducible isolation of dCS with a MW of 12.6 ± 0.6 kDa, and a PD of 1.41 ± 0.05. To our knowledge, this is the first time that CS samples of meaningful quantities—enough for further study and experiment—were reported to be isolated based on their MW.

Furthermore, it was determined that the selection of soluble dCS at a neutral pH is not enough to guarantee a fully soluble dCS in pure water. Both the removal of ions and phase change (from liquid to solid) of the dCS solutions were responsible for the formation of irreversible aggregated dCS particles in water. The addition post dialysis and lyophilization of sodium chloride salt in water was not efficient to reverse the crystallized dCS.

The solid form of a product is the most convenient and often most stable form for packaging and shipping [44,45] for industrial applications. Regarding potential CS-derived drug applications, the results presented herein would suggest that great attention will have to be given to the change in crystallinity of CS-based formulations. In order to ensure the selection of dCS samples which remain fully soluble in pure water independently of phase changes, successive freezing and thawing cycles might offer a valuable technique. 

## Figures and Tables

**Table 1:** Comparison of the average molecular weights of chitosan (CS) and depolymerized chitosan (dCS) measured by gel permeation chromatography (GPC).

Entry	Sample	Mp	Mn	Mw	PD	Heating Conditions	Yield	% DD	% Mn Decrease
1	CS-A	191,500	90,000	253,500	2.82	-	-	85	-
2	CS-B	282,500	174,500	392,000	2.25	-	-	99	-
3 ^c^	dCS-A	n.d.	n.d.	n.d.	n.d.	(80°, 48 h) ^a^	50%	100	
4 ^c^	dCS-B	n.d.	n.d.	n.d.	n.d.	(80°, 48 h) ^a^	49%	100	
5 ^d^	dCS-B	n/a	n/a	n/a	n/a	(100°, 19’) ^b^	<1%	99	95
6	dCS-A	13,600	12,600	17,700	1.41	(100°, 19’) ^b^	15%	85	86

^a^ Conventional heating. ^b^ Microwave-assisted heating. ^c^ GPC analysis was not conducted on dCS resulting from conventional heating. ^d^ GPC analysis could not be performed due to the very low depolymerization yield.

**Table 2:** Progressive dissolution of CS-A, CS-B and dCS-A in aqueous acidic solution.

Entry	Sample	Starting pH ^a^	Final pH ^b^
1	CS-A	6.40	2.19
2	CS-B	7.48	1.93
3	dCS-A	6.86	5.97

Measurements conducted at 23 °C and at a concentration of 10 mg/mL. ^a^ pH obtained after the dissolution of each polymer in water at 10 mg/mL concentration. ^b^ pH reached for the complete solubilization of each polymer in water after the slow addition of 1M HCl.

**Table 3:** Average molecular weights of the dCS sample purified with dialysis over 3 days using membranes of 14, 7 and 3.5 kDa MWCO.

Sample	Mp	Mn	Mw	PD	Yield
dCS (14)	16,100	15,100	19,700	1.31	10%
dCS (7)	16,600	15,000	20,500	1.37	14%
dCS (3.5)	14,200	13,400	18,100	1.36	15%

**Table 4:** Average molecular weights of the dCS samples neutralized to various pH levels and dialyzed in a 7 MWCO dialysis membrane.

Entry	Sample ^a^	Mp	Mn	Mw	PD	Yield	% DD
1	dCS (prec pH 6.7)	41,900	32,800	54,800	1.67	4%	86
2	dCS (prec pH 6.8)	36,300	29,300	50,900	1.74	12%	83
3	dCS (prec pH 6.9)	35,500	28,400	48,200	1.70	18%	84
4	dCS (prec pH 7.0)	33,700	28,700	46,900	1.64	12%	82
5	dCS (prec pH 7.4)	28,200	24,500	38,500	1.57	9%	86
6 ^b^	dCS (sol pH 7.4)	15,100	12,900	18,800	1.46	19%	85

^a^ The samples were neutralized to the pH indicated in parenthesis and the precipitate fraction was isolated and dialyzed at each step for analysis. ^b^ Fraction of dCS remaining in solution when the pH reached 7.4.

**Table 5:** Average concentration of the fully-soluble dCS-A in pure water collected as the sample went through freeze–thaw cycles. Values represent an average of four samples.

Sample	mg/mL
dCS-A (sol I) ^a^	0.22 ± 0.02
dCS-A (cycle 0) ^b^	0.19 ± 0.02
dCS-A (cycle I) ^b^	0.16 ± 0.02
dCS-A (cycle II) ^b^	0.16 ± 0.02
dCS-A (cycle III) ^b^	0.15 ± 0.02
dCS-A (cycle IV–VIII) ^b^	0.15 ± 0.01
dCS-A (sol II) ^c^	0.15 ± 0.01

^a^ dCS-A sample left in solution at 25 °C over 20 days. ^b^ dCS-A samples submitted to freeze–thaw cycles. ^c^ Fraction of dCS-A that remained in solution after eight freeze–thaw cycles.

**Table 6:** GPC analysis of the dCS-B fractions obtained from the dialysis and lyophilization or from centrifugation prior to dialysis.

Sample	Mp	Mn	Mw	PD	DD
dial-dCS-B	7600	8900	12,100	1.36	99%
prec-dCS-B	8500	11,000	15,100	1.38	99%
liq-dCS-B	6800	7200	8700	1.20	99%

## Supplementary Materials

The following are available online at https://www.mdpi.com/2073-4360/12/6/1274/s1, NMR data for dCS-A, Table S1: Effect of time on microwave-assisted depolymerization of CS-A, Table S2: Effect of temperature on microwave-assisted depolymerization of CS-A; Table S3: Effect of concentration on microwave-assisted depolymerization of CS-A, Table S4: Conditions tested for microwave-assisted depolymerization of CS-B, Table S5: Error range of GPC values, Figure S1: Error range of GPC values, Figure S2: Decrease in molecular weight and PD dependence on pH, Figure S3: Solubility of CS-A, CS-B and dCS-A in DMSO.
