# Supplementary material for: Selection of Water-Soluble Chitosan by Microwave-Assisted Degradation and pH-Controlled Precipitation
Source: Polymers (Basel). 2020 Jun 2;12(6):1274. doi: 10.3390/polym12061274 (PMC7362083; doi:10.3390/polym12061274)
Supplement: Supplementary file 1 [file polymers-12-01274-s001.pdf]

Supporting Information

Selection of Water-Soluble Chitosan by Microwave-assisted Degradation and pH Controlled Precipitation

Céline M. A. Journot [1], Laura Nicolle [1], Yann Lavanchy [2] and Sandrine Gerber-Lemaire [1,*]

[1] Group for Functionalized Biomaterials, Institute of Chemical Sciences and Engineering, Ecole Polytechnique Fédérale de Lausanne, EPFL SB ISIC SCI-SB-SG, Station 6, CH-1015 Lausanne, Switzerland;
[2] Molecular and Hybrid Materials Characterization Center, Ecole Polytechnique Fédérale de Lausanne, EPFL STI MHMC MHMC-GE, Station 12, CH-1015 Lausanne

* E—mail : sandrine.gerber@epfl.ch

Table of contents

NMR data for dCS-A S-3

Table S1: Effect of time on microwave-assisted depolymerization of CS-A S-4

Table S2: Effect of temperature on microwave-assisted depolymerization of CS-A S-5

Table S3: Effect of concentration on microwave-assisted depolymerization of CS-A S-6

Table S4: Conditions tested for microwave-assisted depolymerization of CS-B S-7

Table S5 and Figure S1: Error range of GPC values S-8

Figure S2: Decrease of molecular weight and PD dependence on pH S-9

Figure S3: Solubility of CS-A, CS-B and dCS-A in DMSO S-10

NMR analysis of dCS-A

^{1}H NMR (B) (400 MHz, D_2O + 1% DCl) δ 4.89 (s, H1), 4.63 (s, H1'), 4.00-3.49 (m, H3-H6), 3.19 (s, H2), 2.07 (s, H7).

^{1}H NMR (C) (400 MHz, D_2O) δ 4.65 (s, H1), 4.15-3.53 (m, H3-H6), 2.91 (s, H2), 2.07 (s, H7).

Effect of time on microwave-assisted depolymerization of CS-A

Table S1. Effect of time on microwave-assisted depolymerization of CS-A at 100 °C.

Entry	Mp	Mn	Mw	PD	yield	conditions
1	13900	12900	16400	1.28	7%	(100°, 15')
2	15000	13800	18200	1.32	10%	(100°, 17')
3	14300	13100	17800	1.36	15%	(100°, 19')
4	13200	12500	15900	1.28	9%	(100°, 21')
5	12100	11400	14900	1.31	11%	(100°, 23')

The investigation of the effect of time on the depolymerization of CS-A showed that the best yield (15%) was obtained after 19 minutes of microwave-assisted heating (entry 3). GPC analyses indicated that the molecular weight and PD were not significantly affected by the reaction time.

Effect of temperature on microwave-assisted depolymerization of CS-A

Table S2. Effect of temperature on microwave-assisted depolymerization of CS-A (19 minutes).

Entry	yield	conditions
1	2%	(80°, 19')
2	16%	(100°, 19')
3	15%	(120°, 19')[a]
5	15%	(180°, 19')[a]

[a]Formation of degradation by-products identified by the obtention of dark solution and particles.

Effect of concentration on microwave-assisted depolymerization under (100°, 19′) condition of CS-A

Table S3. Variation of the initial concentration of CS-A in microwave-assisted depolymerization (100°, 19').

Entry	yield	concentration
1	13%	0.5%
2	15%	1%
3	14%	2%[a]
4	-	4%[b]
5	-	6%[b]

[a]Pressure inside the reactor increased to 1.8 bar. [b]Pressure inside the reactor increased to 10 bars, resulting in the interruption of the microwave-assisted heating programme.

Conditions tested for microwave-assisted depolymerization of CS-B

We observed that CS-B resisted to microwave-assisted depolymerization with less than 1% yield recovered. Table S1 lists the conditions tested. Protocol for entry 7 is described below. Note that for depolymerization attempted at 120°C (entries 5 and 6), the pressure was observed to increase to 2.2 and 4.2 bars respectively.

Table S4. Conditions tested (time in minutes and temperature in °C) under which CS-B showed resistance to microwave-assisted degradation. Yields were too low (less than 1%) to recover useful amounts of product.

Entry	Conditions
1	(100°, 19')
2	(100°, 30')
3	(100°, 40')
4	(100°, 90')
5	(120°, 60')
6	(120°, 90')
7*	(100°, 90+90')

Protocol entry 7: The depolymerization was done in two steps. The first 90 minutes-depolymerization was done as described in the material and method section (section 2.2), adapting the time from 19 to 90 minutes. No increase of pressure was observed during the microwave treatment. After separation of the solution from the precipitate, the liquid fraction was dialyzed as usual. The precipitate was washed 2 additional times by adding 40 mL of deionized water to the Eppendorf tube, shaking it to re-suspend the precipitate followed by centrifugation. The precipitate was then lyophilized before being dissolved in 20 mL of fresh 1M HCl. The fresh acid solution was applied to the same depolymerization cycle (i.e: 100 °C for 90 minutes) and treated as before. The liquid solution recovered from both 90-minutes depolymerization were combined (after dialysis) and lyophilized together. The amount of dCS-B recovered this way was too small to be fully analyzed and used for further work.

Error range of GPC values

The reproducibility of the microwave-assisted depolymerization method for (100°, 19′) was assessed by repeating the protocol multiple times on CS-A and analyzing the resulting samples by ^{1}H NMR and GPC. We were able to show that a variation of approximately 5% of the molecular weight is observed between the different batches of dCS (Table S5 standard deviation and Figure S1). A yield of approximately 15% was also observed to be consistent between the experiments. No noticeable variation was detected on the ^{1}H NMR spectrum; see Figure 1 (C) for typical spectra of dCS-A.

Table S5. GPC, yield and DD values of dCS-A prepared by microwave-assisted heating at 100 °C for 19 minutes in 1M HCl. Entries 5 and 6 show the standard deviation and average of the GPC analysis.

Entry	Mp	Mn	Mw	PD	yield	DD
1	12900	12100	17700	1.47	20%	85%
2	13200	12100	17300	1.44	13%	85%
3	14100	13400	17100	1.35	15%	84%
4	14300	13100	17800	1.36	16%	85%
st. deviation	600	600	285	0.05	-	-
average	13600	12600	17500	1.41	-	-

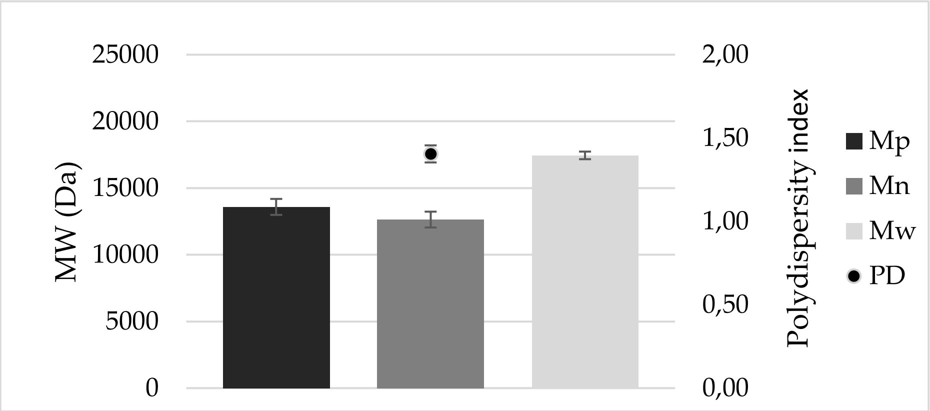

Figure S1. Graphical representation for the values from Table S5. Standard deviation is shown as the error bars.

Decrease of molecular weight and PD dependence on pH

We investigated the effect of the pH at which the crude dCS solution was neutralized and the speed at which the given pH was reached (Table 4). Both parameters were shown to influence the MW and the yield of the final polymer. The selection of the soluble fraction at pH 7.4 led to an average MW approximately 50% smaller (from Mn = 24490 to Mn = 12850), as well as an improvement of the PD (from 1.57 to 1.46). Stepwise neutralization, including a 45 min waiting time before centrifugation, allowed improving the yield by approximately 5%.

We showed that collection of the soluble fraction between two pH values allowed the selection of intermediate MW and a decrease of the PD value as the pH increased above 7.0 (Figure S2). It is interesting to note that the PD value starts by increasing between pH 6.7 and 6.8, and then decreases until the maximum pH is reached. As no more precipitate formed at pH 7.4, no further selection of soluble fraction could be isolated above this pH.

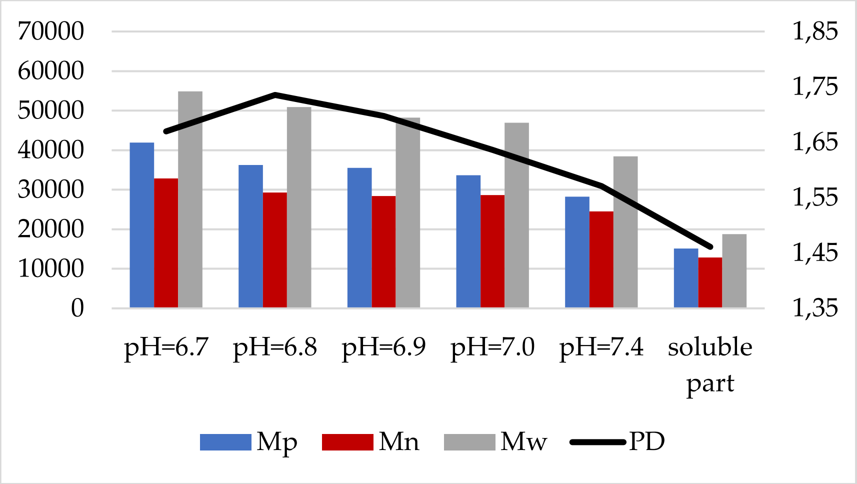

Figure S2. Quantitative representation of Table 4. Isolation and analysis of precipitates as pH increased stepwise showed that the average MW of the dCS fraction decreases, and that the PD also improved (i.e: decreased) for pH 7.0 and higher.

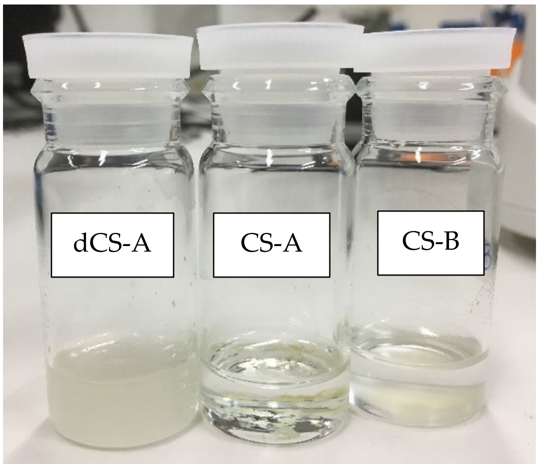

Figure S3. Solubility of CS-A, CS-B and dCS-A in DMSO (22 °C, 6.7 mg/mL). Picture taken after 10 min at rest.
